# New Model for Couple Therapy for Patients with Chronic Pain and their Caregivers: An Attempt to Improve Quality of Life and Reduce Pain

**DOI:** 10.2174/1745017902016010053

**Published:** 2020-06-21

**Authors:** Shima Rouhi, Payman Dadkhah, Manijeh Firoozi, Masoud Hashemi

**Affiliations:** 1 Department of Clinical Psychology, University of Tehran, Tehran, Iran; 2 Department of Anesthesiology, Fellowship in Pain Management, Shahid Beheshti University of Medical Sciences, Tehran, Iran; 3 Department of Health Psychology, University of Tehran, Tehran, Iran; 4 Program Director of Pain Fellowship, Shahid Beheshti University of Medical Sciences, Tehran, Iran

**Keywords:** Chronic pain, Psychological treatment, Acceptance and commitment therapy, Mindfulness, Quality of life, Pain

## Abstract

**Background:**

Several psychological interventions have been implemented to manage chronic pain. In this study, in addition to the patients, his/her spouses have participated in the program. Besides, this innovative therapy integrates several practical approaches into one comprehensive protocol.

**Objective:**

This study aimed to analyze the effectiveness of couple therapy (patient/caregiver-oriented) on improving the quality of life and reducing pain among patients with chronic pain.

**Methods:**

The present study is a quasi-experimental and clinical trial with a control group with pretest and posttest. The authors conducted this study at LABAFINEJAD Hospital in Tehran on 30 patients with chronic pain and their spouses by having a short form of a questionnaire for quality of life and chronic pain score questionnaire to measure the effectiveness of the treatment.

**Results:**

The results indicated that this treatment increased two aspects of quality of life remarkably, social function and strength for continuing the performance; that help boosts interpersonal relationships as well. Regarding the results, although the couple-based treatment could improve all aspects of pain, the two primary subscales, physical health and mental health, both enhanced. Besides, the treatment reduced the intensity of pain.

**Conclusion:**

Couple-based intervention through increasing social support, improving the quality of sex, decentralizing of pain, and paying attention to the neglected needs of caregivers and patients with chronic pain can improve quality of life and reduce pain in patients.

## 1 INTRODUCTION

Chronic pain is one of the most significant health problems involved in the medical system [1]. Continuous pain disturbs a person’s ability to perform their daily activities, such as to be effective in society and the family and to maintain an independent lifestyle [2]. Also, this health problem costs the individual, the family, and the community. At the individual level, unmanageable pain can reduce the quality of life and the level of efficiency, and increase interpersonal problems [3]. Several studies have shown that chronic pain hurts the quality of life and predisposes the patient with chronic pain to a wide range of psychological disorders such as anxiety, depression [4], and sleep disorder [5]. In the previous studies also, chronic pain has been shown to reduce the quality of life in all aspects, especially two general components of physical health and mental health. The quality of life has a bilateral relationship with the quality of sexual activity and supporting interactions with the spouse. [6]. Therefore, if the relationships between the patient-caregiver improve, the quality of life of patients may increase. The results of the research showed that the severity of pain affects the ability to withstand the shock and continue daily activities. Thus, the patient’s dependence is closely monitored by the authors [7]. Dependence on caregivers generates long-lasting negative mutual emotions. A reluctance to get out of dependency, since the patient is unable to do his/her mundane duties, brings out negative feelings such self-denying. These adverse feelings raise conflicts between a couple, the caregiver and patient, ultimately cause marital dissatisfaction and loss of social protection [8]. Research has shown that interactions between couples can predict the physical health of spouses [9]. For couples having a good relationship, it is feasible to cope with the stress associated with chronic pain and non-verbal reactions [10]. Also, a healthy relationship between spouses can predict mental health and care behaviours. Whereas, spouse’s critique and anger-like action can increase the severity of pain [11]. A study found that in women living with chronic illness, and whose husbands expressed sympathetic responses, in the long run, showed better physical performance than those who responded with less sensitivity [12]. Psychological interventions focused on the quality of life of patients with chronic pain are widespread. However, studies that measure their effectiveness have confirmed the impact of low or moderate individual therapies [11]. Patients with pain usually suffer from a lot of interpersonal problems; neglecting the issues or employing personalized psychological treatments will make them less likely to benefit. Therefore, stepping forward to invent an intervention which may improve interpersonal relationships is a compelling goal to enhance the quality of life of patients with CP. The new approach is a combined approach with a couple of strategies that were used to improve the quality of life of patients with chronic pain and their spouses, who have a caring role. The techniques of this method were based on the combination of the most useful therapies for pain management [13]. This therapeutic approach consists of combining treatment approaches based on acceptance and commitment therapy, mindfulness, mindfulness-based on stress reduction, and the use of short-term cognitive-behavioural treatment. The primary purpose of this intervention is to increase the flexibility, and the ability to interact with the spouse effectively. The values and expectations of both participants in the treatment are measured for the patient’s care needs. Previously, to improve interpersonal communication, the caregiver was only used as a therapist’s assistant for instructing strict health orders. Nevertheless, in the new approach, the caregiver is involved actively in the intervention as a participant, not an instructor. The main feature of this intervention is such that couples actively participate in the course of treatment, and both have personal benefits and ultimately share satisfaction [14]. To the researcher’s knowledge, no study has yet examined the impact of this treatment on the quality of life of patients with chronic pain and their spouses. Besides, every psychological therapy in the context of different cultures affects differently. Doing such research is also helpful for researchers who seek intercultural effects. The main objective of this study was to evaluate the effectiveness of couple-based therapy and to improve the quality of life of patients with chronic pain.

## 2 OBJECTIVES

This study aimed to examine the effectiveness of a new therapeutic approach, to improve the quality of life, and to reduce the pain intensity of patients with chronic pain.

## 3 METHODS

The present study is a quasi-experimental and clinical trial with a control group with pre-test and post-test. This study has been conducted by researchers at LABAFINEJAD Hospital in Tehran on 30 patients with chronic pain and their spouses. The sampling method was convenience; at first, people who met the criteria of the study were selected from the list of patients. In the second stage, researchers have randomly divided patients into intervention and waiting lists. The inclusion criteria included the diagnosis of chronic pain, being married, and having a minimum secondary education for both couples. Exclusion criteria included having a dramatic personality disorder (categorized in cluster B) such as borderline or histrionic personality disorder, receiving other psychological synchronous treatments. Before the intervention, the patients were informed of therapeutic targets. Their satisfaction was driven to participate in the research. However, almost all patients did not give up attending the treatment sessions. On average, they have participated in six sessions; as required, depending on the circumstance, the number of courses could be in the range of five to seven. The intervention was carried out for both couples simultaneously in each session [1].

### 3.1 Instruments

#### 3.1.1 Intervention Procedure

*First session*: The therapist initiates rapport and obtains information about the relationship, the history of pain, through a semi-structured interview. The familiarity of each of the couples with the logic of intervention- Introducing mindfulness and action based on values. *Second session:* introducing additional mindfulness skills: bring their focus into their body scan meditation and be in the present moment. They learn how to concentrate on each part of their body organs; it started from toes. Identify potential benefits from anniversary exercise- Form the benefits and assess the extent to which each person lives with his or her values- Introducing psychological flexibility and its meaning. *Third session:* Determine specific behaviours and goals in line with values- Definition of mindfulness skills by practicing being in the moment- and be conscious. *Fourth session:* couple based practice of mindfulness-awareness identification and problem solving based on the values and objectives of Behavior-Adaptation of psychological flexibility skills for interpersonal interaction that includes mindful listening and response along with the transfer of positive emotions to the spouse. *Fifth session:* Detecting and solving problems based on the values and objectives of behaviour-psychological flexibility skills for interpersonal relationships and practising psychological flexibility skills. *Sixth session:* Combining the skills learned during the intervention – identifying psychological and interpersonal flexibility skills to continue practicing after the treatment has finished – problem-solving challenges that may patients encounter.

#### 3.1.2 Quality of Life Questionnaire (SF-36)

Ware and Sherburne [15] prepared a short form of Health Survey Questionnaire (SF-36) based on physical and mental components. The questionnaire has 56 questions and eight health-related subscales. The subscales are social function (10 items), the role of physical function (4 items), bodily pain (2 items), general health (5 items), vitality (4 items), social function (2 questions), the role of emotional service (3 issues), and mental health (5 issues). Also, the questionnaire assesses the health status of each person and compares his/her previous year. The survey includes 149 items, which were amassed on 22 000 people, responding to less than 10 minutes in patients with different medical conditions.

Moreover, at the same time, it has excellent reliability. Three separate studies confirmed the validity and reliability of this test. The Cronbach’s alpha scale ranged from 0.73 to 0.93, and the internal consistency coefficient for the eight sub-scales was reported to be 0.76 to 0.95. In Iranian society, this test was performed on 404 students. The ratio of test re-test was 0.79, and Cronbach’s alpha was 0.85 [16].

#### 3.1.3 Chronic Von Corfu Pain Rating Questionnaire

The questionnaire was developed in 1992 by Von-Korff *et al*. for measuring the severity of chronic pain. On this scale, three axes are evaluated, including the severity of pain, the stability or duration of pain, and the degree of pain disability. The respondents respond to each of the seven questions, on a scale with 11 points (0-10), marks the most suitable answer upon their circumstances. The score is estimated in three sub-scales, including pain intensity, the count of disability, and degrees or levels of disability. This questionnaire includes seven questions. Patients provided information on the severity of the pain and the amount of perturbation they experienced during the past six months in their daily activities. Cronbach’s alpha of disability and pain intensity was 0.87 and 0.68, respectively [17]. In this study, these coefficients are 0.92 and 0.85, respectively.

#### 3.1.4 Pain Rating Questionnaire

 The questionnaire provides seven questions about the severity of the pain and the degree of disorder that the patients have been engaged in their daily activities during the past six months. The Cronbach’s alpha was 0.87 and 0.68 for disability and severity of pain, respectively [18]. In this study, these coefficients were 0.92 and 0.85, respectively.

## 4 RESULTS

Table **1** presents the demographic characteristics of patients with chronic pain based on gender, age, and education. The mean age of the subjects in the experimental group was 52.38 and in the control group was 54.86

As shown in Table **1**, out of a total of 16 test groups, six had less than a diploma, 6 were associated degrees, and 4 were bachelor’s degrees. Also, a total of 16 controls among participants, which underwent the intervention (8 individuals) were men, and 50% of those were women_ Considering gender proportion. Among patients in the waiting list, 36.4% (9 individuals) were women, and 73.5% (5 individuals) were men.

Table **2** the mean and standard deviation of the factors contributing to the quality of life of patients have reported.

As shown in Table **2**, the most significant change among the subscales of quality of life scale was the social function score. After participating in the treatment, participants indicated the enormous leap from 34.37 in the pre-test to 118.75 in the post-test. At the same time, in the control group, the grades were steady with trivial changes (in pre-test it was 41.28 and in post-test was 57.14).

To determine the changes in the quality of life dimensions, in the two groups, MANCOVA was used (Table **3**). Table **3** shows that there was a significant difference between the experimental and control groups in terms of physical health (*P* = 0.001; *P* = 0.001) and psychological well-being (*P* = 0.001, *P* = 0.79).

The mean score of pain intensity in the experimental group was 46.5 in pre-test, while after undergoing the treatment, this amount was reduced to 29.5 at post-test. At the same time, in the control group the pain intensity was constant (pre-test was 47 and post-test was 47.5)

Chart (**1**) shows changes in the quality of life scores in both physical and psychological dimensions.

The results of MANCOVA on the mean scores of the posttest show the amount of pain in the studied groups with pretest control.

Based on the results, the severity of pain after the intervention was significantly decreased (*P*= 0.71).

## 5 DISCUSSION

The present study showed that couple therapy (focused on chronic pain) improved the quality of life of patients with chronic pain and reduced the amount of perceived pain.

Hoffman *et al*. [18], in a meta-analysis, reviewed how psychological intervention improves the physical and mental symptoms of patients with pain. They found that, apart from the treatment methods used, the studies that had been used by the control group yielded more accurate results. Without treatment used, the effect of size was significant for the quality of life and perceived pain severity, and other variables had a negligible effect. Similar studies have led us to consider the most sensitive variables, namely quality of life and pain intensity, as a measurement of the effectiveness of treatment. The results also showed that psychological interventions had an impact on these two variables. Vieehof *et al*. [14], in another meta-analysis, examined the effect of the size of psychological variables after psychological interventions for chronic pain. They also found that the most significant impact of treatments was on the treatment of Acceptance and Commitment Therapy (ACT) and the Mindfulness-Based Stress Reduction (MBSR). They found that the severity of pain and the quality of life of patients with chronic pain were more than anxiety and depression. Several studies emphasized that acceptance and commitment and mindfulness therapy had a modest effect on improving psychological symptoms. However, their role in reducing pain and improving the quality of life was significant [18]. In the psychosocial treatment of the couple (patient-caregiver), a combination of ACT and MBSR was used by researchers. The results of the study, in coherence with the results of other studies, show improvement in the quality of life and pain reduction. Bergeron *et al*. [19] compared a series of psychological interventions, cognitive-behavioural skills training, and pair-training skills for chronic pain. They found that the learning of cognitive-behavioural skills led to improve cognitive and physical abilities, but the couple therapy aims at enhancing interpersonal relationships. This finding was inconsistent with the results of this study. The present study showed that the betterment of the emotional state of caregivers could improve both physical and psychological dimensions' patients. Cano *et al*. [20] showed that couple-based therapy affected chronic pain to reduce helplessness and distress. They found that when both couples were involved in exercising flexion, adaptive pain was more than the time when the only patient has intervened. Kindt *et al*. [21] showed that when collaboration between a pair, one of whom with chronic pain, was raised, the independence of the patient would increase, the quality of the couple's relationship would improve, and anxiety and helplessness would decrease. These findings, in line with the results of this study, confirm the effectiveness of coupled interventions in improving physical and psychological performance. A couple-based treatment can affect the quality of life of patients with pain in several ways; firstly, empowering the caregiver increases his/her mental capacity to support the patient. A practitioner can be considered that is under pressure in any way to interact with the fatigue and anger patient; these negative emotions increase the severity of pain and discomfort in the patient [22]. In a couple-based treatment, the goal is to increase flexibility and to reduce stress. Therefore, the caregiver also finds the opportunity to manage his emotions correctly. Besides, the patient also exercises latitude. Negative emotions can also reduce the patient's pain. Less pain affects the marital relationship, as well. Secondly, this method of treatment is effective in improving the quality of the relationships between couples, which makes them begin a more favourable sexual relationship, and those who have stopped it because of the pain, have an opportunity to restart it again. A pleasant sexual relationship with endorphin secretion will reduce pain.

Thirdly, changes in the attitude of couples about pain as the uncontrollable issues can be useful, and they try to restrain it. As a consequence, they are, instead of blaming each other, or being angry resulting from the pain, looking for an efficient solution to manage it. They can throw away a vain attempt to stop the pain. It seems that the key to effectiveness and excellence in this therapeutic approach is to increase mutual social support. In this method, the focus has shifted from the patient individually to the patient and caregiver's needs, which fade into oblivion. The limitation of this research is the absence of a follow-up period and a modest number of participants.

## CONCLUSION

The results showed that social function and energy for the continuation of the performance had improved the most. According to the results, although the couple-based therapy could improve all aspects of physical and psychological subscales of pain, the physical health of patients was principally affected. This finding indicates that dealing with deep-rooted psychological problems requires more sessions; indeed, more than six-session treatment have had a positive impact on physical function. It is beneficial to carry out future research with the follow-up period and more participants. For future studies, it is suggested to emphasize cultural aspects that enhance couples' interpersonal relationships. Each culture has special features to enhance couples' ability to struggle with chronic pain. Researchers also suggest that therapeutic techniques should adjust to the elderly, who are more likely to be associated with comorbid illnesses and pain experience.

## Figures and Tables

**Chart (1):**
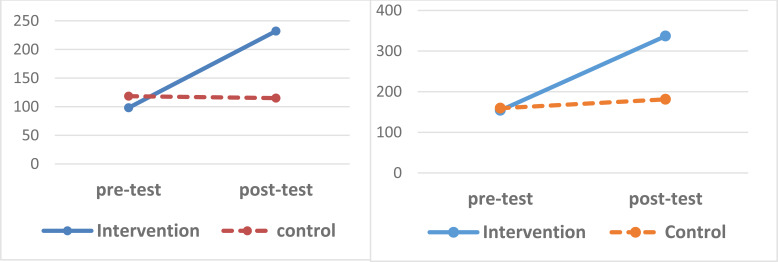
Changes in quality of life scores in both physical and psychological dimensions The scores of later physical health quality of life in the studied groups. Sores of mental health scores. Quality of life in the studied groups

**Table 1:** Demographic characteristics of patients with chronic pain.

**Intervention**	**Control**	**Sub-variables**	**Variables**
8 (50%)	9 (64.3%)	Woman	**Gender**
8 (50%)	5 (35.7%)	Man	
52.38 (11.11)	54.86 (11.1)		**Age**
6 (37.5%)	7 (50%)	L.D	**Education**
6 (37.5%)	2 (14.3%)	A	
4 (25%)	4 (28.6%)	B.D	
0 (0%)	1 (7.1%)	M.A	

L.D= less than diploma, A= associated degree, BD= bachelor's degree, M.A= master degree

**Table 2:** The mean and standard deviation of subscales of quality of life scores in intervention groups and waiting lists.

**Post-test**	**Pre-test**	**Groups**	**Variables**
M±SD	M±SD		
62.18±20.08	34.06±29.84	Intervention	**Physical-function**
32.14 ± 27.92	32.86± 31.18	Control	
67.18± 21.83	17.19 ± 29.88	Intervention	**Role of physical function**
23.21 ± 31.72	19.69 ± 31.24	Control	
79.17 ± 31.91	33.33 ± 43.88	Intervention	**Role of emotional dysfunction**
28.57 ± 36.64	26.26 ± 29.77	Control	
69.37 ± 9.28	41.25 ± 24.46	Intervention	**Vitality**
15.54 ± 45.71	18.02 ± 43.57	Control	
69.5 ± 12.88	45 ± 26.14	Intervention	**Emotional well-being**
49.17 ± 18.07	48.28 ± 20.42	Control	
118.75 ± 28.14	34.37 ± 22.59	Intervention	**Social function**
57.14 ± 33.15	41.28 ± 21.59	Control	
47.03 ± 17.44	20 ± 21.60	Intervention	**Bodily-pain**
28.21 ± 21.82	29.11 ± 25.61	Control	
55.62 ± 15.26	26.87 ± 17.4	Intervention	**General-health** **Control**
31.43 ± 18.23	36.78 ± 18.67	Control	

**Table 3:** Summary of the results of one-way covariance analysis in the MANCOVA text on the mean posttest scores of quality of life dimensions.

**Average difference**	**P**	**P**	**F**	**MS**	**D.F**	**Sum of Squares**	**Scales**
1	0.84	0.0001	135.49	124461.62	1	124461.62	**Physicalhealth**
1	0.79	0.0001	96.72	177007.07	1	177007.7	**Mentalhealth**
1	0.77	0.0001	87.78	2291.09	1	2291.09	**Pain intensity**

MS, mean of squares;

## References

[1] McCracken L.M., Vowles K.E. (2008). A prospective analysis of acceptance of pain and values-based action in patients with chronic pain.. Health Psychol..

[2] Huntoon M.A. (2017). Pain Medicine Board Review..

[3] Williams A.C.C., Craig K.D. (2016). Updating the definition of pain.. Pain.

[4] Weisbord S.D. (2016). Patient‐centered dialysis care: Depression, pain, and quality of life.. Seminars in dialysis..

[5] Elbinoune I., Amine B., Shyen S., Gueddari S., Abouqal R., Hajjaj-Hassouni N. (2016). Chronic neck pain and anxiety-depression: prevalence and associated risk factors.. Pan Afr. Med. J..

[6] Almoznino G., Benoliel R., Sharav Y., Haviv Y. (2017). Sleep disorders and chronic craniofacial pain: Characteristics and management possibilities.. Sleep Med. Rev..

[7] Mapplebeck J.C., Beggs S., Salter M.W. (2017). Molecules in pain and sex: a developing story.. Mol. Brain.

[8] Mohammadi S., Dehghani M., Sanderman R., Hagedoorn M. (2017). The role of pain behaviour and family caregiver responses in the link between pain catastrophising and pain intensity: A moderated mediation model.. Psychol. Health.

[9] Liu J-F., Lu M.C., Fang T.P., Yu H.R., Lin H.L., Fang D.L. (2017). Burden on caregivers of ventilator-dependent patients: A cross-sectional study.. Medicine (Baltimore).

[10] Cano A., Tankha H. (2018). Spousal criticism and hostility in response to pain: what is the alternative?. Pain.

[11] Roditi D., Robinson M.E. (2011). The role of psychological interventions in the management of patients with chronic pain.. Psychol. Res. Behav. Manag..

[12] Berry E., Davies M., Dempster M. (2017). Exploring the effectiveness of couples interventions for adults living with a chronic physical illness: A systematic review.. Patient Educ. Couns..

[13] Cano A., Corley A.M., Clark S.M., Martinez S.C. (2018). A couple-based psychological treatment for chronic pain and relationship distress.. Cognit. Behav. Pract..

[14] Veehof M.M., Oskam M.J., Schreurs K.M., Bohlmeijer E.T. (2011). Acceptance-based interventions for the treatment of chronic pain: a systematic review and meta-analysis.. Pain.

[15] Tunis S.L., Croghan T.W., Heilman D.K., Johnstone B.M., Obenchain R.L. (1999). Reliability, validity, and application of the medical outcomes study 36-item short-form health survey (SF-36) in schizophrenic patients treated with olanzapine versus haloperidol.. Med. Care.

[16] Gh S. (2015). Psychometric characteristics of the health survey questionnaire (SF-36) for evaluation of multiple sclerosis: psychometric evaluation in Iranian patients.. Majallah-i Ilmi-i Pizishki-i Jundi/Shapur.

[17] Von Korff M., Dworkin S.F., Le Resche L. (1990). Graded chronic pain status: an epidemiologic evaluation.. Pain.

[18] Hoffman B.M., Papas R.K., Chatkoff D.K., Kerns R.D. (2007). Meta-analysis of psychological interventions for chronic low back pain.. Health Psychol..

[19] Bergeron S. (2018). Couple sex therapy versus group therapy for women with genito-pelvic pain.. Curr. Sex. Health Rep..

[20] Cano A., Goubert L. (2017). What’s in a name? The case of emotional disclosure of pain-related distress.. J. Pain.

[21] Kindt S., Vansteenkiste M., Loeys T., Cano A., Lauwerier E., Verhofstadt L.L., Goubert L. (2015). When is helping your partner with chronic pain a burden? The relation between helping motivation and personal and relational functioning.. Pain Med..

[22] Miller-Matero L.R., Cano A. (2015). Encouraging couples to change: A motivational assessment to promote well-being in people with chronic pain and their partners.. Pain Med..

